# Supplementary material for: Exposures to Potentially Psychologically Traumatic Events among Canadian Coast Guard and Conservation and Protection Officers
Source: Int J Environ Res Public Health. 2022 Nov 16;19(22):15116. doi: 10.3390/ijerph192215116 (PMC9690280; doi:10.3390/ijerph192215116)
Supplement: Supplementary file 1 [file ijerph-19-15116-s001.zip › ijerph-2029821-supplementary.pdf]

Supplementary Table S1. Prevalence of Potentially Psychologically Traumatic Events (PPTEs) Exposure Types- Expanded Table

Type of Exposure	Total Sample	CCG	C&P	PSP [4]	Comparing Prevalence among Total Sample and PSP	General Population [7]	Comparing Prevalence among Total Sample and General Population [7]	General Population [6]	Comparing Prevalence among Total Sample and General Population [6]
	% (*n*)	% (*n*)	% (*n*)	% (*n*)	Test Statistics	%	Test Statistics	%	Test Statistics
Life threatening natural disaster (e.g., flood, hurricane, tornado, earthquake, and wildfires)									
Ever exposed[1]	65.0(268)	66.9(186)	74.8(80)	66.4 (2,832)	-0.53	15.6	27.59***	7.7	43.57***
1 to 5 times	86.1(211)	88.4(153)	80.6(58)	91.7 (2,376)					
6 to 10 times	6.5(16)	5.8(10)	8.3(6)	3.5 (90)					
11 or more times	7.3(18)	5.8(10)	11.1(8)	4.8 (125)					
Happened to me	40.7(109)	38.7(72)	46.3(37)	-					
Witnesses it	37.7(101)	33.9(63)	47.5(38)	-					
Learned about it	43.7(117)	45.2(84)	38.8(31)	-					
Part of my public safety job	31.0(83)	29.0(54)	36.3(29)	-					
Fire or explosion				-					
Ever exposed	68.9 (284)	73.4(204)	72.9(78)	86.0 (3,727)	-9.91***	n/a	-	n/a	-
1 to 5 times	83.4(216)	84.4(157)	80.8(59)	50.4 (1,805)					
6 to 10 times	5.0(13)	5.9(11)	^	10.1 (361)					
11 or more times	11.6(30)	9.7(18)	16.4(12)	39.6 (1,419)					
Happened to me	30.3(84)	27.9(57)	34.6(27)	-					
Witnesses it	43.3(123)	39.7(81)	53.8(42)	-					
Learned about it	40.1(114)	40.2(82)	38.5(30)	-					
Part of my public safety job	39.1(111)	43.1(88)	29.5(23)	-					
Serious transportation accident (e.g., car accident, boat accident, train wreck, plane crash)				-					
Ever exposed	77.4(319)	81.3(226)	85.0(91)	93.2 (4,084)	-12.62***	17.8	31.58***	14.0	37.03***
1 to 5 times	68.0(210)	65.3(143)	74.4(67)	22.7 (907)					
6 to 10 times	7.4(23)	5.9(13)	11.1(10)	6.0 (241)					

11 or more times	24.6(76)	28.8(63)	14.4(13)	71.3 (2,845)					
Happened to me	35.1(112)	30.5(69)	47.3(43)	-					
Witnesses it	44.5(142)	40.3(91)	56.0(51)	-					
Learned about it	37.3(119)	36.3(82)	38.5(35)	-					
Part of my public safety job	47.6(152)	47.8(108)	48.4(44)	-					
Serious accident at work, home, or during recreational activity				-					
Ever exposed	69.7(287)	73.4(204)	75.7(81)	81.6 (3,430)	-6.19***	7.9	46.38***	6.2	53.31***
1 to 5 times	86.6(232)	86.5(167)	86.7(65)	66.6 (2,091)					
6 to 10 times	5.2(14)	5.2(10)	^	6.5 (205)					
11 or more times	8.2(22)	8.3(16)	8.0(6)	26.9 (844)					
Happened to me	35.5(102)	34.8(71)	38.3(31)	-					
Witnesses it	49.5(142)	46.6(95)	58.0(47)	-					
Learned about it	39.0(112)	38.2(78)	39.5(32)	-					
Part of my public safety job	33.8(97)	30.4(62)	43.2(35)	-					
Exposure to toxic substance (e.g., dangerous chemicals, radiation)				-					
Ever exposed	50.0(206)	53.2(148)	52.3(56)	67.4 (2,664)	-7.48***	10.1	26.80***	4.2	46.22***
1 to 5 times	71.7(132)	68.6(94)	80.9(38)	61.3 (1,439)					
6 to 10 times	4.9(9)	5.8(8)	^	7.4 (174)					
11 or more times	23.4(43)	25.5(35)	17.0(8)	31.3 (735)					
Happened to me	43.7(90)	45.3(67)	41.1(23)	-					
Witnesses it	25.2(52)	26.4(39)	23.2(13)	-					
Learned about it	40.3(83)	33.8(50)	55.4(31)	-					
Part of my public safety job	56.8(117)	60.1(89)	50.0(28)	-					
Physical assault (e.g., being attacked, hit, slapped, kicked, beaten up)				-					
Ever exposed	69.4(286)	69.8(194)	84.1(90)	90.6 (3,931)	-14.65***	9.2	42.20***	5.9	54.61***
1 to 5 times	75.0(189)	76.0(46.8)	72.8(59)	41.8 (1,543)					
6 to 10 times	8.7(22)	7.6(13)	11.1(9)	9.5 (350)					
11 or more times	16.3(41)	16.4(28)	16.0(13)	48.7 (1,797)					
Happened to me	61.9(177)	60.8(118)	65.6(59)	-					
Witnesses it	46.2(132)	42.8(83)	54.4(49)	-					
Learned about it	40.6(116)	39.7(77)	41.1(37)	-					
Part of my public safety job	28.0(80)	17.0(33)	52.2(47)	-					

Assault with a weapon (e.g., being shot, stabbed, threatened with a knife, gun, bomb)				-					
Ever exposed	52.7(217)	48.6(135)	74.8(80)	83.9 (3,639)	-17.18***	16.0	20.24***	14.5	21.93***
1 to 5 times	92.2(153)	94.1(95)	89.2(58)	57.6 (1,797)					
6 to 10 times	^	^	^	9.4 (294)					
11 or more times	6.0(10)	^	9.2(6)	32.9 (1,027)					
Happened to me	36.9(80)	34.8(47)	41.3(33)	-					
Witnesses it	25.8(56)	25.9(35)	26.3(21)	-					
Learned about it	55.8(121)	55.6(75)	55.0(44)	-					
Part of my public safety job	28.1(61)	18.5(25)	45.0(36)	-					
Sexual assault (e.g., rape, attempted rape, made to perform any type of sexual act through force or threat of harm)				-					
Ever exposed	52.4(216)	55.8(155)	55.1(59)	71.2 (3,035)	-8.52***	11.4	26.13***	5.8	40.39***
1 to 5 times	91.7(143)	91.8(112)	91.2(31)	47.1 (1,089)					
6 to 10 times	^	^	^	11.0 (255)					
11 or more times	5.8(9)	4.9(6)	-	41.9 (968)					
Happened to me	35.2(76)	38.1(59)	28.8(17)	-					
Witnesses it	8.8(19)	8.4(13)	10.2(6)	-					
Learned about it	70.8(153)	70.3(109)	71.2(42)	-					
Part of my public safety job	9.7(21)	8.4(13)	13.6(8)	-					
Other unwanted or uncomfortable sexual experience				-					
Ever exposed	59.2(244)	64.7(180)	57.9(62)	67.3 (2,803)	-3.44***	21.9	18.26***	4.2	55.56***
1 to 5 times	72.0(152)	72.5(116)	72.0(36)	55.2 (1,225)					
6 to 10 times	5.2(11)	5.0(8)	^	7.7 (171)					
11 or more times	22.7(48)	22.5(36)	22.0(11)	37.2 (825)					
Happened to me	63.9(156)	69.4(125)	48.4(30)	-					
Witnesses it	18.0(44)	17.8(32)	17.7(11)	-					
Learned about it	45.9(112)	40.6(73)	59.7(37)	-					
Part of my public safety job	12.3(30)	11.7(21)	14.5(9)	-					
Combat				-					
Ever exposed	22.6(93)	21.9(61)	28.0(30)	18.8 (791)	1.90*	4.3	18.16***	3.2	22.20***
1 to 5 times	73.1(38)	71.4(25)	76.5(13)	78.4 (349)					
6 to 10 times	^	^	^	21.6 (96)					
11 or more times	23.1(12)	22.9(8)	-	-					

Happened to me	16.1(15)	21.3(13)	^	-					
Witnesses it	14.0(13)	11.5(7)	20.0(6)	-					
Learned about it	76.3(71)	70.5(43)	86.7(26)	-					
Part of my public safety job	17.2(16)	21.3(13)	^	-					
Captivity (e.g., being kidnapped, abducted, held hostage, prisoner of war)				-					
Ever exposed	15.5(64)	14.7(41)	19.6(21)	30.5 (1,279)	-6.55***	1.4	24.21***	1.1	27.85***
1 to 5 times	92.5(37)	96.2(25)	85.7(12)	78.9 (712)					
6 times or more	-	-	^	21.2 (191)					
11 or more times	^	^	-	-					
Happened to me	12.5(8)	14.6(6)	^	-					
Witnesses it	10.9(7)	12.2(5)	^	-					
Learned about it	82.8(73)	78.0(32)	90.5(19)	-					
Part of my public safety job	^	2.4(1)	^	-					
Life threatening illness or injury				-					
Ever exposed	66.5(274)	69.8(194)	72.9(78)	76.7 (3,301)	-4.84***	32.0	14.96***	11.8	34.34***
1 to 5 times	83.4(206)	84.4(152)	80.3(53)	54.2 (1,594)					
6 times or more	5.3(13)	4.4(8)	7.6(5)	6.3 (184)					
11 or more times	11.3(28)	11.1(20)	12.1(8)	39.6 (1,165)					
Happened to me	27.0(74)	29.4(57)	21.8(17)	-					
Witnesses it	57.3(157)	52.6(102)	67.9(53)	-					
Learned about it	44.5(122)	40.2(78)	55.1(43)	-					
Part of my public safety job	27.0(74)	28.9(56)	23.1(18)	-					
Severe human suffering				-					
Ever exposed	53.2(219)	56.5(157)	56.1(60)	79.1 (3,234)	-12.89***	3.4	55.59***	3.7	53.05***
1 to 5 times	70.8(126)	66.4(87)	82.6(38)	41.8 (1,187)					
6 times or more	7.3(13)	9.2(12)	^	6.2 (177)					
11 or more times	21.9(39)	24.4(32)	15.2(7)	51.9 (1,473)					
Happened to me	10.0(22)	12.1(19)	^	-					
Witnesses it	49.8(109)	44.6(70)	61.7(37)	-					
Learned about it	47.0(103)	43.9(69)	55.0(33)	-					
Part of my public safety job	35.6(78)	39.5(62)	26.7(16)	-					
Sudden violent death (e.g., homicide, suicide)				-					
Ever exposed	65.8(271)	69.1(192)	72.0(77)	93.8 (4,101)	-23.49***	n/a	-	n/a	-
1 to 5 times	80.9(195)	78.8(134)	85.9(61)	36.4 (1,426)					
6 times or more	6.6(16)	5.9(10)	8.5(6)	13.1 (512)					

11 or more times	12.4(30)	15.3(26)	^	50.5 (1,977)					
Happened to me	9.2(25)	10.9(21)	^	-					
Witnesses it	27.3(74)	22.4(43)	40.3(31)	-					
Learned about it	65.7(178)	63.0(121)	71.4(55)	-					
Part of my public safety job	33.2(90)	35.9(69)	27.3(21)	-					
Sudden accidental death				-					
Ever exposed	69.4(286)	73.4(204)	74.8(80)	93.7 (4,063)	-20.19***	41.1	11.63***	1.4	117.30***
1 to 5 times	78.5(194)	76.1(137)	85.1(57)	34.6 (1,321)					
6 times or more	6.9(17)	7.2(13)	^	10.7 (408)					
11 or more times	14.6(36)	16.7(30)	9.0(6)	54.8 (2,092)					
Happened to me	8.4(24)	8.8(18)	7.5(6)	-					
Witnesses it	36.4(104)	31.9(65)	48.8(39)	-					
Learned about it	56.6(162)	52.9(108)	65.0(52)	-					
Part of my public safety job	39.9(114)	43.1(88)	32.5(26)	-					
Serious injury, harm, or death you caused to someone else				-					
Ever exposed	13.8(57)	12.9(36)	19.6(21)	36.2 (1,485)	-9.40***	n/a	-	26.3	-5.69***
1 to 5 times	80.0(28)	81.0(17)	78.6(11)	64.4 (580)					
6 times or more	^	-	^	6.8 (61)					
11 or more times	17.1(6)	^	^	28.9 (260)					
Happened to me	31.6(18)	30.6(11)	33.3(7)	-					
Witnesses it	43.9(25)	47.2(17)	38.1(8)	-					
Learned about it	42.1(24)	30.6(11)	61.9(13)	-					
Part of my public safety job	31.6(18)	33.3(12)	28.6(6)	-					
Any other very stressful event or experience				-					
Ever exposed	31.1(128)	30.6(85)	39.3(42)	-	-	8.6	16.18***	40.8	-3.97***
1 to 5 times	72.1(98)	69.7(62)	78.3(36)	-					
6 times or more	8.1(11)	9.0(8)	^	-					
11 or more times	19.9(27)	21.3(19)	15.2(7)	-					
Happened to me	69.5(89)	68.2(58)	71.4(30)						
Witnesses it	28.1(36)	27.1(23)	28.6(12)						
Learned about it	19.5(25)	16.5(14)	23.8(10)						
Part of my public safety job	50.8(65)	54.1(46)	45.2(19)						
**Total number of different types of potentially traumatic exposures, *Mean* (*SD*)**	8.72(4.42)	9.05(3.87)	9.76(4.07)	11.08 (3.23)	*t*(411)=-10.85***	2.31 (2.33)	*t*(411)=29.41***	2.2-3.7	-

Notes: CCG= Canadian Coast Guard; C&P=Conservation and Protection; PSP=public safety personnel; *SD*=Standard Deviation; n/a=not available.

[1] Not all individuals who report ever being exposed reported the number of exposures. So, the number of exposures does not necessarily match ever exposed.
^Sample size between 1 and 5, so data not presented. $^{*}p<.05$, $^{**}p<.01$, $^{***}p<.001$ – Statistically significantly different at these p-values.
